# Comparison of Biaxial Biomechanical Properties of Post-menopausal Human Prolapsed and Non-prolapsed Uterosacral Ligament

**DOI:** 10.1038/s41598-020-64192-0

**Published:** 2020-04-30

**Authors:** Elvis K. Danso, Jason D. Schuster, Isabella Johnson, Emily W. Harville, Lyndsey R. Buckner, Laurephile Desrosiers, Leise R. Knoepp, Kristin S. Miller

**Affiliations:** 1 Department of Biomedical Engineering, Tulane University, 6823 St. Charles Ave, New Orleans, LA 70118 USA; 2 Department of Epidemiology, Tulane University, 1440 Canal Street, Suite 2000, New Orleans, LA 70112 USA; 3 Department of Research, Biorepository Unit, Ochsner Health System, 1514 Jefferson Highway, New Orleans, LA 70121 USA; 4 Department of Female Pelvic Medicine & Reconstruction Surgery, Ochsner Clinical School, 1514 Jefferson Highway, New Orleans, LA 70121 USA

**Keywords:** Diseases, Risk factors

## Abstract

Uterosacral ligaments (USLs) provide structural support to the female pelvic floor, and a loss of USL structural integrity or biomechanical function may induce pelvic organ prolapse (POP). Alterations in extracellular matrix composition and organization dictate USL mechanical function. Changes in USL microstructure and corresponding mechanical properties, however, are not fully understood, nor is it understood how microstructure and mechanics change with onset and progression of POP. This is due, in part, as USL properties are primarily characterized along a single direction (uniaxial test), whereas the USL is loaded in multiple directions simultaneously within the body. Biaxial testing permits the acquisition of biomechanical data from two axes simultaneously, and thus simulates a more physiologic assessment compared to the traditional uniaxial testing. Therefore, the objective of this study was to quantify the biaxial biomechanical properties and histological composition of the USL in post-menopausal women with and without POP at various stages. Potential correlations between tissue microstructural composition and mechanical function were also examined. Tangential modulus was lower and peak stretch higher in POP III/IV compared to non-POP and POP I/II in the main *in vivo* loading direction; however, no significant differences in mechanical properties were observed in the perpendicular loading direction. Collagen content positively correlated to tangential modulus in the main *in vivo* loading direction (*r* = 0.5, *p* = 0.02) and negatively correlated with the peak stretch in both the main *in vivo* (*r* = *−*0.5, *p* = 0.02) and perpendicular loading directions (*r* = −0.3, *p* = 0.05). However, no statistically significant differences in USL composition were observed, which may be due to the small sample size and high variability of small sections of human tissues. These results provide first step towards understanding what microstructural and mechanical changes may occur in the USL with POP onset and progression. Such information may provide important future insights into the development of new surgical reconstruction techniques and graft materials for POP treatment.

## Introduction

Pelvic organ prolapse (POP) is characterized by the abnormal descent of female pelvic organs due to the loss of structural support, often resulting in defecatory and urinary symptoms, and sexual dysfunction^1,2^. In the United States, approximately 12% of women undergo surgical intervention to repair anatomical defects and relieve symptoms due to POP^3^. Unfortunately, as many as 30% of these surgical interventions fail, due to insufficient strength of native tissue or mismatches in the mechanical and structural properties and maladaptive tissue remodeling associated with implanted grafts, and require re-operation^4^. Additionally, a direct cost of over one billion dollars is incurred annually for POP-associated interventions in the United States^5^. Furthermore, knowledge of POP pathophysiology is still limited and the etiology of POP is not fully elucidated, although risk factors include mechanical injury, vaginal delivery, obesity, age, race, family history, higher parity, constipation and menopausal status^6–10^.

The pelvic floor has multiple levels of support^6,11^. Level I support comprises the cardinal-uterosacral ligament (USL) complex, which provides apical attachment of the vaginal vault and uterus to the sacrum. Level II support comprises the arcus tendinous and fascia of the levator ani, which provide support to the mid vagina. The lower vagina is supported by the urogenital diaphragm and the perineal body, comprising level III support. Although the levator ani muscles play a vital role in protecting the pelvic connective tissues from the impact of excessive load and support the pelvic organs^12–14^, not all women with levator ani injury develop POP. Likewise, not all women with POP are diagnosed with levator ani tears^13,15^.

As previously mentioned, the USLs provide Level I support to the female pelvic floor^16^. Changes in the extracellular matrix composition and organization dictate pelvic floor mechanical function^17^. Therefore, changes in USL extracellular matrix composition and organization may lead to a loss of biomechanical and structural function thereby inducing POP or POP progression. The USL is primarily composed of collagen fibres, smooth muscle cells (SMC), and a small quantity of elastic fibres^16,18,19^. Relationships between USL extracellular matrix composition and biomechanical function, however, are not fully elucidated, as the female pelvic floor is drastically understudied from a biomechanical perspective. Primarily uniaxial tests are conducted to quantify the biomechanical function of the USL^20–23^. While these tests provide important information, the USL is loaded multiaxially within the body, thus, biaxial mechanical properties are critical in order to understand changes in USL function that occurs with POP and POP progression^24^. Planar biaxial testing permits assessment of tissue anisotropy and the acquisition of biomechanical data from two axes simultaneously, providing a relevant physiologic assessment^25–27^. Such information is necessary to develop synthetic or biomaterial grafts with similar mechanical properties to the adjacent native tissues to minimize graft complications^28^. While recent work characterized USL biaxial biomechanical function in swine and women without POP^25^, the changes in human prolapsed tissue remain unknown.

Pelvic organ prolapse can be categorised into five stages (0–4) based on a standard measuring tool, the Pelvic Organ Prolapse Quantification (POP-Q) system^29^. The POP-Q was developed to enhance both academic and clinical communication for individuals as well as larger populations^29,30^. Additionally, it provides a standard measuring system to facilitate the process of understanding relationships between anatomy and function, and between interventions and anatomy^30^. According to the POP-Q system, the absence of POP is defined Stage 0. In Stage I, the leading point is >1 cm above the level of hymen; in Stage II, the leading edge of prolapse lies within 1 cm above and 1 cm below the level of the hymen; in Stage III, the leading edge of prolapse is at >1 cm below the hymen and no further than 2 cm less than the total vaginal length (TVL); and, Stage IV describes prolapse with leading edge >TVL-2 cm and includes complete vaginal vault eversion or procidentia uteri^29,31,32^. Changes in mechanics and microstructural composition of the USL as a function of POP stage are unknown. Further, menopause, the permanent termination of menstruation due to loss of ovarian follicular function^33^ is a risk factor for development of POP^34^. In fact, nearly 51% of women at menopause have anterior wall prolapse^35^. Therefore, the objective of this study was to quantify the biaxial biomechanical properties of the USL in post-menopausal women with and without POP. Additionally, tissue microstructure was characterized to determine potential relationships between microstructure and mechanical function. We hypothesised that (1) USLs in the main *in vivo* loading direction would demonstrate increased material stiffness compared to the perpendicular direction, (2) USLs from women with POP would demonstrate lower tangential modulus and collagen content compared to non-POP controls, and that (3) collagen and elastic fibre content would correlate to tangential modulus and peak stretch.

## Methods

**Figure 1:**
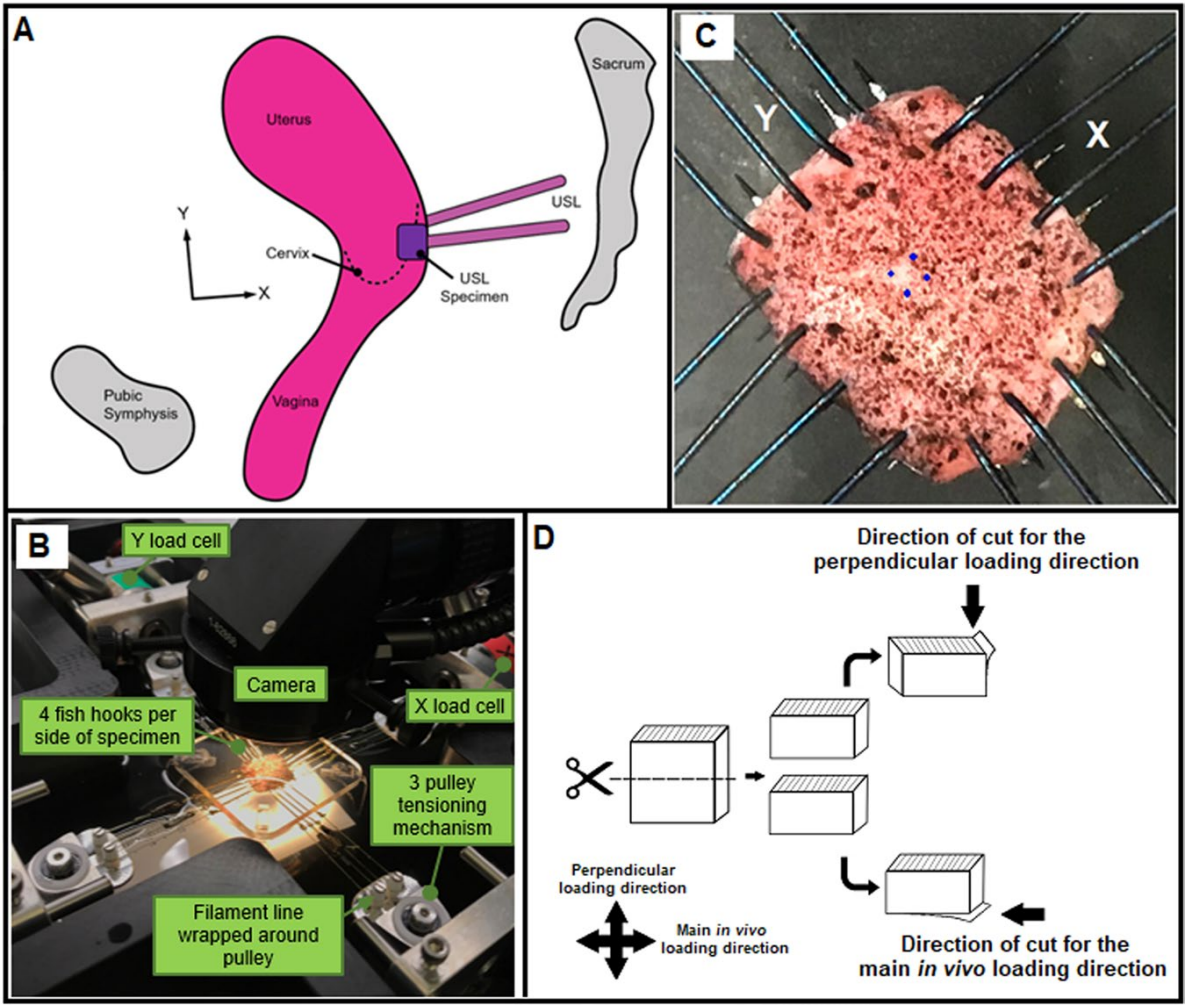
Specimens were obtained at the insertion of USL into the posterior uterine body at the distal right and left locations by trained Urogynecologists (**A**). Custom-built planar biaxial testing device equipped with load cells in both axes and a camera (**B**). USL samples were speckle-coated and mounted in a planar biaxial testing device via fishhooks. Strain was tracked optically in the center of the specimen (4 points shown in blue) (**C**). Histological sections were obtained along the main *in vivo* and perpendicular loading directions for the compositional and structural analyses (**D**).

Samples were obtained from the Ochsner Biorepository Unit with signed pre-operative informed consent from women undergoing hysterectomy. All methods were performed in accordance with relevant local guidelines and regulations. Experimental protocols were approved by the relevant local Institutional Review Board (Ochsner Health System IRB approved: 2017.016A). Prior to surgical intervention, two Fellowship-trained Urogynecologists at Ochsner Medical Center in New Orleans, LA determined the POP-Q stage. Menopausal status, the permanent termination of menstruation for more than one year, was collected from the patient’s clinical chart/medical record. During surgery, the aforementioned Fellowship-trained Urogynecologists excised bilateral USL samples located at the insertion into the uterine body from post-menopausal women (Fig. 1A). Immediately following excision, the surgeons marked the USL samples with a suture to denote the perpendicular loading direction to ensure sample orientation was consistent during mechanical and histological assessment. Next, samples were snap-frozen and stored at −80 °C until the day of biomechanical assessment. All patient records were de-identified by Ochsner Biorepository Unit personnel. Research personnel were blinded to patient case status and demographics during mechanical and histological analyses to prevent bias.

For patient recruitment, the inclusion criteria for non-POP controls (*n* = 10) were female, menopause, no symptoms or evidence of prolapse on exam, undergoing hysterectomy for benign indications. The exclusion criteria were male, non-menopausal, cancer diagnosis, connective tissue disorder, previous surgery for pelvic floor disorder, symptomatic prolapse, and hormone replacement therapy (Table 1). The inclusion criteria for the POP cases (*n* = 14) were female, symptomatic prolapse undergoing hysterectomy for benign indications, and the exclusion criteria (male, cancer diagnosis, connective tissue disorder, previous surgery for pelvic floor disorder, and hormone replacement therapy) (Table 1). Samples from patients characterized with POP-Q prolapse stages of I and/or II in any compartment were classified as POP I/II (*n* = 8). POP III/IV (*n* = 6) were samples from patients with POP-Q stages III and/or IV in any compartment^29,30^ (Table 1).

**Table 1:** Patient demographics from which the samples were obtained for the study.

Sample	Age - at time of surgery	BMI [kg/m^2^]	Parity	Gravidity	Race	POP-Q, type and stage of Prolapse; Diagnosis
1	68	31.81	1	1	African-American	No prolapse
2	66	37.84	2	2	African-American	No prolapse
3	57	35.16	2	2	African-American	No prolapse
4	60	27.98	0	0	Caucasian	No prolapse, breast cancer
5	65	36.74	2	2	Caucasian	No prolapse
6	68	39.79	2	2	Caucasian	No prolapse
7	66	27.32	2	3	African-American	No prolapse
8	59	26.45	1	1	Caucasian	No prolapse
9	66	27.3	2	3	African-American	No prolapse
10	52	32.18	3	5	Middle-Eastern	No prolapse
**Mean**	**62.70**	**32.26**				
**SEM**	**1.71**	**1.55**				
1	52	24.03	3	4	Caucasian	Stage 2 apical prolapse, Stage 2 anterior and posterior vaginal wall prolapse
2	52	36.02	4	8	Middle-Eastern	Stage 2 apical prolapse, Stage 2 anterior and posterior vaginal wall prolapse
3	70	22.64	2	2	Caucasian	Stage 2 apical prolapse, Stage 2 anterior and posterior vaginal wall prolapse
4	59	25.35	0	0	Caucasian	Stage1 apical prolapse, Stage 2 anterior and posterior vaginal wall prolapse
5	58	44.99	1	1	Caucasian	Stage 2 cystocele/uterine prolapse
6	59	24.9	1	1	Caucasian	Incomplete Stage 2 cystocele/stage 1 uterine prolapse, breast cancer
7	69	24.69	2	2	Caucasian	Incomplete stage 2 cystocele, stage 1 rectocele
8	67	36.37	2	4	Caucasian	Stage 2 incomplete
**Mean**	**60.75**	**29.87**				
**SEM**	**2.53**	**2.89**				
1	68	26.05	2	4	Caucasian	Stage 3 cystocele/stage 1 uterovaginal prolapse/stage 2 rectocele
2	64	26.34	3	3	Caucasian	Stage 3 cystocele, stage 2 uterine, stage 2 rectocele
3	62	32.34	5	6	Caucasian	Complete Stage 4 apical prolapse
4	74	25.92	2	2	Caucasian	Stage 3 anterior vaginal wall prolapse, Incomplete Stage 2 apical prolapse, breast cancer
5	60	34.94	2	2	Caucasian	Incomplete Stage 3 cystocele/uterovaginal prolapse, stage 2 rectocele
6	75	44.91	1	1	Caucasian	Stage 3 anterior and stage 2 posterior vaginal wall prolapse, Incomplete Stage 2 apical prolapse
**Mean**	**67.17**	**31.75**				
**SEM**	**2.56**	**3.05**				

### Biaxial testing

Prior to the biomechanical test, samples were thawed at room temperature, and cut into squares to ensure consistent sample dimensions and to remove any tissue serosa, charred sections from cauterization, or inhomogeneous portions due to surgical intervention along the main *in vivo* loading direction (X) and the direction perpendicular to that (Y). Sample orientation was denoted by the surgeon’s suture, which was replaced with India ink to denote the perpendicular loading direction. This ensured consistent and repeatable orientation during mechanical testing and subsequent histological analysis (Fig. 1B). Using a non-contacting laser micrometer, tissue thickness was measured across multiple locations and averaged (3.4 ± 0.2 mm). The unloaded width along both directions were also measured with a caliper (7.4 ± 0.2 mm). The minimum length and thickness used were 3.5 mm and 1.3 mm, respectively. The square samples were then speckle coated with alcohol ink (Ranger Industries, Tinton Falls, NJ, USA) to enable optical strain tracking during biomechanical assessment. Four fish hooks were used to mount each sample side (Fig. 1C) into a custom planar biaxial test device equipped with load cells (22 N) in both axes, and a camera^25,27^. The device permits independent control of displacement in the two orthogonal axes. The samples were fully submerged in Hank’s balance saline solution (HBSS) at room temperature throughout the experiment. To prevent movement of HBSS around the mounted sample, an acrylic glass table was placed over the bath, making a complete contact with the HBSS solution as described previously^25^.

A tare load of 0.1 N was applied in the main *in vivo* and perpendicular loading directions, followed by 10 equibiaxial preconditioning cycles to a target stress of 0.1 MPa at 0.2%/sec, after which the samples were allowed to equilibrate for 10 minutes. Next, samples were loaded to a maximum stress level of 0.1 MPa in the main *in vivo* and perpendicular loading directions to different X:Y loading ratios^36–38^ of 1:0.5, 1:0.75, 1:1, 0.75:1 and 0.5:1 at 0.2%/sec, for 6 cycles. The maximum stress was informed by prior studies in the USL that indicated no damage to the underlying tissue microstructure during mechanical assessment^26,39^. A loading ratio of 1:1, denotes an equibiaxial loading to the maximum stress of 0.1 MPa, whiles 1:0.5 denotes a maximum stress of 0.1 MPa in the main *in vivo* loading direction and 0.05 MPa in the perpendicular loading direction. Each loading ratio lasted ~15–20 minutes. Therefore, samples were subjected to mechanical testing for approximately 100 minutes for the 5 loading ratios herein, as well as 5 additional protocols for pilot studies - total ~200 minutes. The experiments were terminated following the completion of the 6^th^ cycle (once the sample was returned to the original tare load). Data from the 6th unloading cycle was used for the analyses.

**Figure 2:**
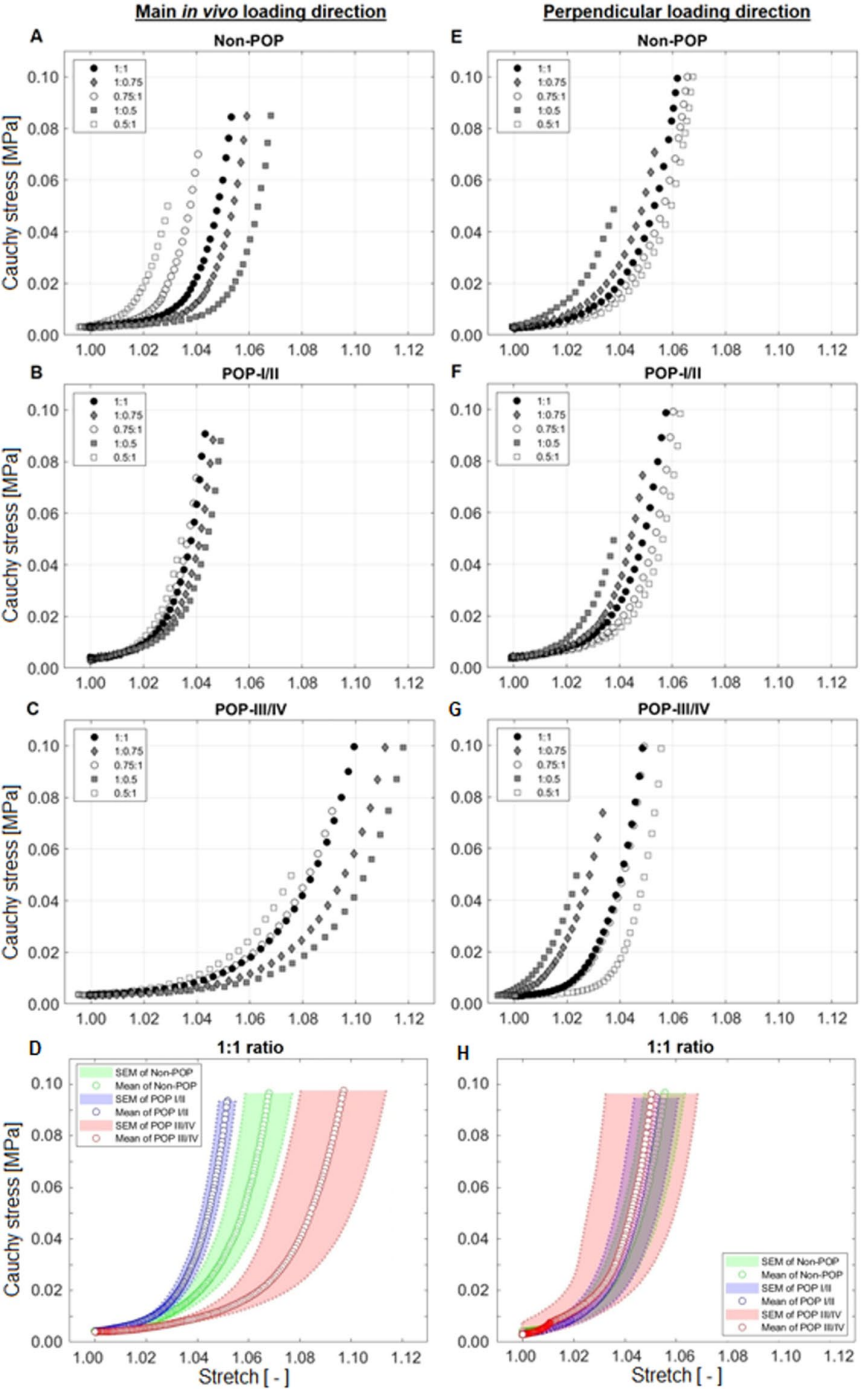
Representative Cauchy stress-stretch plots of various loading ratios of USL in the main *in vivo* loading direction (left column) for non-POP (**A**), POP I/II (**B**) and POP III/IV (**C**) specimens. Representative Cauchy stress-stretch plots of various loading ratios of USL in the perpendicular loading direction (right column) for non-POP (**E**), POP I/II (**F**) and POP III/IV (**G**) specimens. USL exhibited anisotropic behaviour for non-POP, POP I/II and POP III/IV. Mean (SEM) of Cauchy stress-stretch plots of USL loaded at 1:1 ratio for non-POP and various POP stages (I-IV), in the main *in vivo* (**D**) and perpendicular (**H**) loading directions. Figure shows that, in the main *in vivo* loading direction, POP III/IV exhibited more extensibility compared to non-POP and POP I/II. There were, however, no differences in extensibility observed in the perpendicular loading directions. Green, blue and pink colours are for non-POP, POP I/II and POP III/IV USL, respectively.

Using a custom-written Matlab script, the images were exported to GOM Correlate, a commercially available software (GOM GmbH, Braunschweig, Germany, v2.0.1). This software enabled tracking of selected speckle points for non-contact strain measurement. Four points in the central portion of the sample (when connected form a quadrilateral), away from the tissue edges were selected for tracking (Fig. 1C). The displacement components in the main *in vivo* and perpendicular loading directions of the four selected locations were obtained and saved as CSV files. Similar to other soft biological tissues, the USL’s mechanical response was nonlinear (Fig. 2). Thus, a phenomenological Fung constitutive model was used to describe the experimental data due to low stiffness at small strains, followed by high stiffness at higher strains^36,40^. A custom-written Matlab script was then used to calculate the deformation gradient tensor $\,({\bf{F}})\,$from the displacements of the selected speckle points^41^. The first Piola-Kirchhoff stress tensor **P** (in both main *in vivo* and perpendicular loading directions) was calculated from the measured axial load in one direction divided by the undeformed cross-sectional area of the sample. Using Nanson’s formula, and with the assumption of incompressibility ($J={\rm{\det }}\,{\bf{F}}=1$)^37,41^, Cauchy stress, ***σ*** was then determined from the relation:

1 $${\boldsymbol{\sigma }}={J}^{-1}{\bf{P}}{{\bf{F}}}^{T}$$

It was assumed that the USL maintains its volume during deformation (incompressibile). The Fung-type exponential pseudostrain-energy function is given by:

2 $$W=\frac{1}{2}K({e}^{Q}-1)$$

where$$\,Q={c}_{1}{E}_{xx}^{2}+{c}_{2}{E}_{yy}^{2}+2{c}_{3}{E}_{xx}{E}_{yy}.$$

The model parameter $K$ represents a stress-like material parameter that describes the bulk tissue resistance to deformation. Model parameters $\,{c}_{1}$, ${c}_{2},\,{c}_{3}$ are dimensionless and describe the direction-dependent resistance to deformation. Model parameters $\,{c}_{1}$ and ${c}_{2}$ indicate the biomechanical contributions from the collagen fibres aligned in the main *in vivo* and perpendicular directions, respectively. Model parameter $\,{c}_{3}\,$represents the potential interaction between collagen fibres aligned in the main *in vivo* and perpendicular loading directions – thus may include physical phenomena such as crosslinks or connections between the collagen fibres to each other or to the ground matrix^42^. $\,{E}_{xx}$, ${E}_{yy}\,$are Green strains in the main *in vivo* and perpendicular loading directions, respectively^42^.

The in-plane Green-Lagrange strain tensor $\,({\bf{E}})$ for each direction was then calculated using

3 $${\bf{E}}=\frac{1}{2}({{\bf{F}}}^{T}{\bf{F}}\,-\,{\bf{I}}),\,$$

where ${{\bf{F}}}^{T}{\bf{F}}={\bf{C}}\,$is the right Cauchy-Green deformation tensor, and ${\bf{I}}$ is the second-order identity tensor.

With the assumption of incompressibility, the theoretical Cauchy stress tensor was determined by^43^:

4 $${\bf{t}}=-p{\bf{I}}+2{\bf{F}}\frac{\partial W({\bf{C}})}{\partial {\bf{C}}}{{\bf{F}}}^{T}.\,$$

where $p$ is a Lagrange multiplier that enforces incompressibility (${\rm{\det }}\,{\bf{F}}=1$)

Using a custom-written Matlab code and a built-in minimization algorithm (*lsqnonlin*), the Fung-type constitutive model was used to describe the experimental data (combined ratios) of the control non-POP and POP cases to determine the direction-dependent nonlinear tissue behaviour by minimizing the objective function

5 $$\mathop{\sum }\limits_{i=1}^{n}[{({\sigma }_{xx}^{th}-{\sigma }_{xx}^{exp})}_{i}^{2}+{({\sigma }_{yy}^{th}-{\sigma }_{yy}^{exp})}_{i}^{2}],\,$$

where $\,{\sigma }_{xx}$ and ${\sigma }_{yy}$ denote the Cauchy stress in the main *in vivo* and perpendicular loading directions respectively and, superscripts $th\,$and $\,exp$ denote theoretically computed and experimentally measured values respectively.

Material anisotropy index, $\phi$ was calculated based on obtained model parameters of Eq. (2)^36,37^ using equation:

6 $$\phi =min\left(\frac{{C}_{1}+{C}_{3}}{{C}_{2}+{C}_{3}},\frac{{C}_{2}+{C}_{3}}{{C}_{1}+{C}_{3}}\right).$$

The material anisotropy index, $\phi$, is a direction-dependent numerical value between 0 and 1 that provides information on the contributions from the main *in vivo* and perpendicular loading directions to the USL stiffness. Specifically, $\,\phi =1$ is an indication that the USL is perfectly isotropic, and smaller values of $\,\phi$ are associated with increasing anisotropy^36,37^. The tangential modulus was calculated as the slope of the linear portion of stress (force/area)-stretch curve^44^. This is a measure of the resistance to deformation of the USL normalized by cross-sectional area (material stiffness). The tangential modulus determined herein serves as a first approximation to a potential target mechanical property for future graft development, in addition to the aforementioned nonlinear properties quantified herein. The peak stretch was determined from the stretch value that corresponded to the maximum stress. This parameter quantified USL extension under the largest applied load.

### Histology

**Figure 3:**
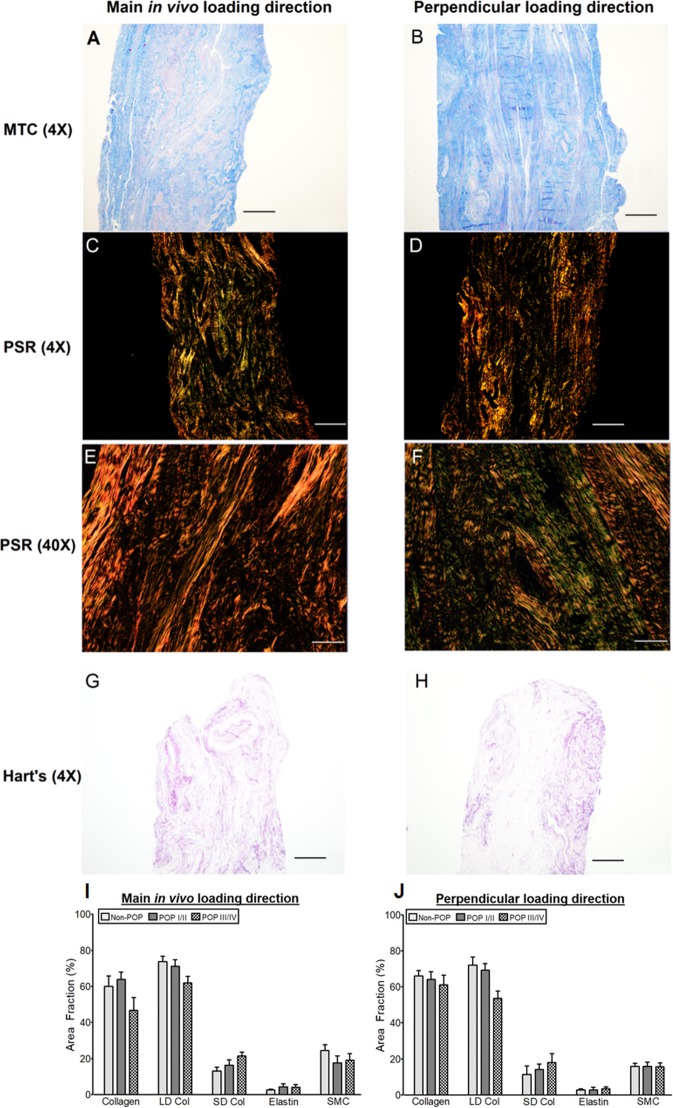
Representative histological samples were obtained in the main *in vivo* (**A,C,E,G**) and perpendicular (**B,D,F,H**) loading directions, stained with Masson’s trichrome (MTC) (**A,B**), Picrosirius red (PSR) (**C**–**F**), and Hart’s elastin (**G,H**). Area fraction analysis were conducted using images obtained at 4X magnification except for collagen structural information (orientation, alignment and straightness ratio) which were analyzed with PSR images obtained at 40X magnification (**E,F**). Scale bars: 200 µm for the images with 40X magnification (**E,F**); 500 µm for all other images (4X magnification) (**A–D, G**–**H**). Mean (SEM) of area fraction of USL composition in the non-POP and POP (I-IV) in the main *in vivo* (**I**) and perpendicular (**J**) loading directions. Non-parametric Kruskal-Wallis test was used for the USL group comparisons. There were no statistically significant differences in both the main *in vivo* and perpendicular loading directions which may be due to the small sample size, lack of specific immunohistochemical or western blot analyses, or analysis of small sections of tissue that may be represent the entire tissue. LD Col=large-diameter collagen, SD Col=small-diameter collagen.

Following mechanical testing, samples were cut in half, and each was randomly allocated for histological analyses along either the main *in vivo* or perpendicular loading direction (Fig. 1D). Each loading direction was marked with a different colour, fixed in a 10% formalin solution for 24 hours, and then embedded in paraffin. Subsequently, 4 µm sections were cut along the main *in vivo* and perpendicular loading directions (Figurer 1D). Thereafter, sections were stained with Masson’s trichrome (MTC), Picrosirius red (PSR), and Hart’s elastin stains (Fig. 3). MTC was used to quantify collagen and SMC, PSR was used to quantify large-diameter and small-diameter collagen fibres, as well as collagen structural information (orientation, alignment and straightness ratio). Hart’s elastin was also used to quantify elastic fibres.

Brightfield (MTC, Hart’s elastin) and darkfield (PSR) images were obtained with an Olympus BX51 microscope, an Olympus DP27 digital camera, and cellSense^TM^ software (Olympus Corporation, Center Valley, PA, U.S.A). For the PSR images, a quarter wavelength retardation plate was used, which converts linearly polarized light input to a circularly polarized output wave front by introducing a relative retardation of exactly one-quarter wavelength or 90 degrees between the ordinary and extraordinary wave front. Throughout PSR imaging, the microscope stage was not rotated. All images were obtained at 4× magnification, and additional PSR images were obtained at 40× magnification (Figs. 3E,F) for the quantification of collagen orientation, alignment, and straightness ratio^17,45^. To quantify the SMC and collagen area fractions, MTC images were analysed with ImageJ’s (National Institutes of Health, Bethesda, MD, USA) open source colour deconvolution plug-in^45,46^ and an open source GNU image manipulation program (GIMP)^45,47–49^. The previously established colour deconvolution used herein is capable of separating the colours in their red, blue and green absorption characteristics^45–47^. The red and blue colours represent the area fractions of SMC and collagen respectively. To quantify the large- and small-diameter collagen, PSR images (4×) were utilized, leveraging a custom-written MATLAB code that calculates the area fractions of red and orange pixels (large-diameter collagen), and yellow and green pixels (small-diameter collagen)^45,50,51^. For the quantification of collagen structure, PSR images (40×) were used. An open source MATLAB software framework that includes two separate but linked packages (CurveAlign and CT-FIRE) was employed^52^. The CurveAlign package was used to quantify the collagen orientation and alignment, while the CT-FIRE was leveraged to quantify straightness ratio. To quantify the elastic fibre area fraction, Hart’s stained images were used, employing GIMP’s select-by-colour tool to isolate elastic fibres^17,45^.

### Statistical analysis

First, the clinical demographics of the patient populations between groups (age, BMI, parity, gravidity) were compared to determine if a correction factor was necessary. Then, comparisons between the USL groups (non-POP, POP I/II and POP III/IV) were performed for mechanics (peak stretch and tangential modulus), Fung model parameters ($K,\,{c}_{1},{c}_{2},\,{c}_{3}$), anisotropic index, composition (collagen, large- and small-diameter collagen, elastic fibre, SMC) and collagen microstructure (orientation, alignment, straightness ratio). First, the data was checked for normality with three assumptions, (1) test of normality by Shapiro-Wilk test, (2) homogeneity of variances by Levene’s test, and (3) detection of outliers. Values more than 3 times the interquartile range (IQR) were considered as outliers using box and whisker plots^53^. When all aforementioned normality assumptions were satisfied, ANOVA was implemented, followed by post-hoc Tukey’s HSD, or an independent samples t-test was used when comparing two groups (risk factor and composition). When the data was not normally distributed, a non-parametric Kruskal-Wallis test (at least 3 groups) or Mann-Whitney U test (2 groups) was implemented.

To identify potential correlations between extracellular matrix microstructural composition (collagen, elastic fibre, SMC, orientation, alignment, straightness ratio), and biomechanical function (peak stretch and tangential modulus) Pearson’s or Kendall’s tau-b correlations were performed if the error was normally or not normally distributed, respectively (Table 2). Further, we investigated potential relationships between POP clinical demographics (age, BMI, parity, gravidity) and histological composition.

**Table 2:** Statistical methods used between composition/structure and biomechanics based on normality of data, homogeneity of variances and outliers. * indicates statistical significance.

	Main *in vivo* loading direction		Perpendicular loading direction	
	Peak Stretch	Tangential modulus	Peak stretch	Tangential modulus
Collagen	Pearson *	Pearson*	Kendall’s tau-b*	Pearson
Large diameter collagen	Pearson	Pearson	Kendall’s tau-b	Kendall’s tau-b
Small diameter collagen	Pearson	Pearson	Kendall’s tau-b	Kendall’s tau-b
Elastin	Kendall’s tau-b	Kendall’s tau-b	Kendall’s tau-b	Kendall’s tau-b
Smooth muscle cell	Pearson	Pearson	Kendall’s tau-b	Pearson
Collagen orientation	Pearson	Pearson	Kendall’s tau-b	Pearson
Alignment	Pearson	Pearson	Kendall’s tau-b	Pearson
Straigtness ratio	Kendall’s tau-b	Kendall’s tau-b	Kendall’s tau-b	Kendall’s tau-b

## Results

### Demographics

No statistically significant differences were identified with respect to age (*p* = 0.17), BMI (*p* = 0.20), parity (*p* = 0.38), and gravidity (*p* = 0.46) between the non-POP, POP I/II, or POP III/IV groups (Table 1).

### Biaxial biomechanical properties

All USL samples exhibited a nonlinear behaviour in both the main *in vivo* and perpendicular loading directions (Fig. 2). POP III/IV USLs were the most extensible when compared to the USLs from non-POP or POP I/II patients in the main *in vivo* loading direction regardless of loading ratio (Fig. 2A–D). Contrastingly, no significant differences were observed in the perpendicular direction (Fig. 2E–H).

**Figure 4:**
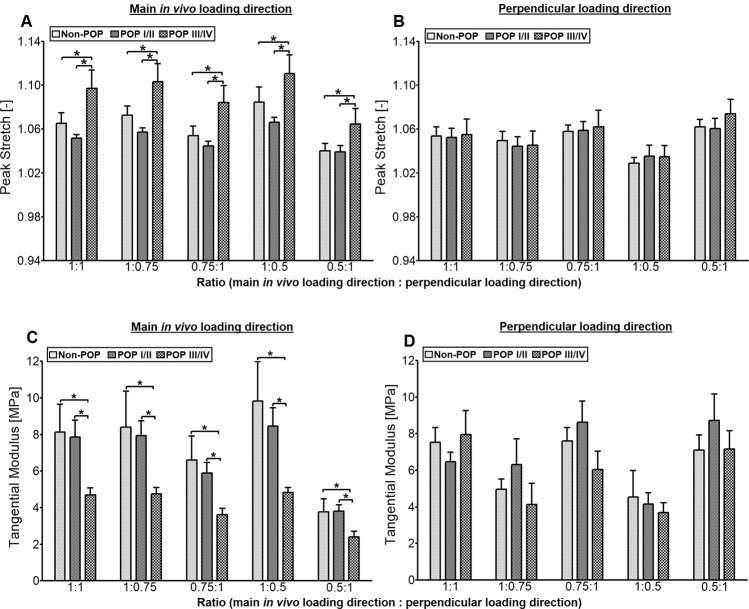
Mean (SEM) of peak stretch and tangential modulus of various loading ratios of USL of non-POP and POP (I-IV) in the main *in vivo* (**A,C**) and perpendicular (**B,D**) loading directions. Three-way ANOVA was implemented with loading direction (main *in vivo* and perpendicular), USL group (non-POP, POP I/II and POP III/IV), and loading ratio (1:1, 1:0.75, 0.75:1, 1:0.5 and 0.5:1) as the factors. There were significant main effects of loading direction (*p* < 0.001, peak stretch) and USL group (*p* < 0.001, peak stretch; *p* = 0.028, tangential modulus). Also, there were significant interactions (*p* < 0.01, peak stretch and tangential modulus) between the loading direction and the USL group, as well as, between the loading direction and the loading ratios. Tukey’s HSD post hoc analyses indicated that POP III/IV was significantly different from non-POP (*p* = 0.005, peak stretch) and POP I/II (*p* = 0.001, peak stretch; *p* = 0.037, tangential modulus) USLs.

Peak stretch was normally distributed; hence, a 3-way ANOVA was used to determine the potential effects of loading direction, POP group, and loading ratio. Significant main effects (*p* < 0.001, MSE = 0.001) of loading direction (F = 23.2) and USL POP group (F = 11.5) were identified (Fig. 4A,B). There was also a significant interaction between loading direction and USL POP group (F = 7.6), as well as between loading direction and loading ratios (F = 11.6). Post hoc analyses using Tukey’s HSD indicated that POP III/IV was significantly different (*p* = 0.001) from both non-POP and POP I/II (Fig. 4A,B).

Tangential modulus was normally distributed. The 3-way ANOVA (loading direction, POP group, loading ratio) showed a significant main effect (*p* = 0.02, MSE = 13.1) of USL POP group (F = 4.0) (Fig. 4C,D). Also, there was a significant interaction between loading direction and USL POP group (F = 4.4, *p* = 0.01), as well as between loading direction and loading ratios (F = 7.7, *p* = 0.001). Post hoc analyses using Tukey’s HSD indicated that POP III/IV was significantly different from both non-POP (0.03) and POP I/II (*p* = 0.04). No other significant differences were observed for the peak stretch and tangential modulus.

### Fung constitutive model parameters

All the Fung model parameters and the anisotropic index values were not normally distributed; therefore, a non-parametric Kruskal-Wallis test was used to evaluate differences between POP groups (Table 3). No statistically significant differences were identified (*p* > 0.05) between the groups for model parameters or the anisotropic index. In general, USL samples from POP I/II women exhibited the highest values for all model parameters, including the anisotropy index, except for the parameter $\,K$ – which describes the global tissue resistance to deformation.

**Table 3:** Best fit values (median (min - max)) of Fung-type constitutive model and the anisotropic index obtained for USL from women with and without POP, loaded biaxially.

	*K* [MPa]	*c*_1_ [—]	*c*_2_ [-]	*c*_3_ [—]	*R*^2^	$\phi$
Non-POP	9E-4 (1.8E-4–2.1)	154.5(0.3–395.7)	131.4 (0.3–583)	7.6 (1.6E-5–112.4)	0.9 (0.6–1)	0.8 (0.3–1)
POP I/II	7.2E-4 (2.1E-4–2.7E-3)	171.3 (120.4–291.5)	198.4 (132.3–327)	34.2 (9.4E-13–149.7)	0.9 (0.8–1)	0.8 (0.6–0.9)
POP III/IV	7.7E-4 (1.4E-4–1.1E-3)	122.5 (43–285.7)	132.4 (51.7–251.2)	20.6 (4.8E-7–107.6)	0.9 (0.8–1)	0.7 (0.4–0.9)

### Constituents of USL

The composition data was not normally distributed; therefore, a non-parametric Kruskal-Wallis test was used to evaluate differences between POP groups. No statistically significant differences (*p* > 0.05) were exhibited between the groups (Fig. 3I,J).

For the structural data, collagen orientation and alignment were normally distributed, therefore, a two-way ANOVA (loading direction, POP) was used. The straightness ratio was not normally distributed. Therefore, a non-parametric Kruskal-Wallis test was used. For the collagen orientation, there were no interaction between loading direction and POP group (Table 4). Collagen orientation was significantly different in POP III/IV compared to POP I/II (*p* = 0.04, Table 4). No statistically significant differences (*p* > 0.05) were observed in alignment and straightness ratio (Table 4).

**Table 4:** Structural information (median (min - max)) of USL from women with and without POP obtained from PSR images at 40X magnification (Fig. 3E,F).

	Main *in vivo* loading direction			Perpendicular loading direction		
	Orientation [°]	Alignment	Straightness ratio	Orientation [°]	Alignment	Straightness ratio
Non-POP	89.9 (51.9–125.1)	0.53 (0.24–0.89)	0.93 (0.92–0.94)	86.1 (55.3–100.9)	0.57 (0.35–0.69)	0.93 (0.93–0.94)
POP I/II	80.4 (43.9–112.7)	0.53 (0.34–0.91)	0.93 (0.93–0.94)	69.5 (50–98.5)	0.53 (0.26–0.85)	0.93 (0.93–0.94)
POP III/IV	98.3 (79.5–128.1)*	0.56 (0.28–0.69)	0.93 (0.93–0.94)	87.9 (64–136.9)*	0.44 (0.15–0.88)	0.93 (0.93–0.94)

An orientation of 90̊ indicates that the collagen fibres are predominantly along the direction of loading. Alignment coefficient of 1 indicate perfectly aligned fibres, and smaller values indicate more randomly distributed fibres. Straightness ratio of 1 indicates perfectly straight fibres, and smaller values indicate increasing fibre waviness^52^. Two-way ANOVA (with loading direction and USL group as factors) indicated that, for the orientation, there were no interactions between loading direction and POP group. Post hoc Tukey’s HSD indicated that POP III/IV was significantly different (*p* = 0.04) from POP I/II in the main *in vivo* and perpendicular loading directions. * indicates statistically significant differences compared to POP I/II.

### Correlations between composition and mechanics

In the main *in vivo* loading direction, collagen content significantly correlated with the tangential modulus (*r* = 0.5, *p* = 0.02) and negatively with the peak stretch in both the main *in vivo* (*r* = *-*0.5, *p* = 0.02) and perpendicular loading directions (*r* = −0.3, *p* = 0.05) (Table 2). No other correlations were statistically significant.

### Correlations between composition and clinical demographics

No statistically significant correlations were observed between the USL composition and clinical demographics (age, BMI, parity and gravidity).

## Discussion

This was the first study, to the authors’ knowledge, to investigate potential relationships between biaxial biomechanical properties and microstructure in USL tissues from pos-tmenopausal women with and without POP. USLs demonstrated non-linear stress-strain relationships and direction-dependent mechanical function. USL peak stretch increased and tangent modulus decreased in stage III/IV POP compared to both non-POP and POP I/II groups in the main *in vivo* loading direction, but no significant differences were observed in the perpendicular direction. Further, a significant positive correlation was identified between collagen content and tangential modulus in the main *in vivo* loading direction. A significant negative correlation was identified between collagen content and peak stretch in both loading directions. However, contrary to our hypothesis, no statistically significant differences in collagen content were observed between POP groups and no significant correlations were identified between USL microstructure and POP risk factors such as BMI and parity. The results herein contribute to the understanding of USL direction-dependent mechanical properties with and without POP. These properties are important to develop new graft materials for POP repair and surgical reconstruction techniques. Results also suggest that it may be important to consider direction-dependent changes in USL microstructure and mechanical content function over time and with POP progression.

The increased peak stretch and decreased tangent modulus observed in the POP III/IV group in the main *in vivo* loading direction suggest that USL mechanical function changes with POP progression which may have implications for pelvic organ support. While the etiology of POP is multifactorial and not fully understood, the observed alterations in the USL suggest that changes in pelvic support tissue mechanical properties may contribute to POP progression. Further, the increased extensibility observed in the POP III/IV group may suggest changes in USL length occur with POP progression due to decreased ability to resist *in vivo* loads, which may have implications for reconstructive surgical planning^25^. While the correlations identified in this study may suggest that increased extensibility is due to decreased collagen content, no significant differences in collagen content were identified between POP groups. This may be due to the small sample size, as prior studies show decreased collagen content in women with POP or stress incontinence^54,55^. Additionally, Gabriel *et al*. (2005) reported lower collagen content than that observed in this study. This may be a limitation of the use of PicroSirius Red to quantify collagen content versus specified immunohistochemistry. However, collagen and smooth muscle content measured in this study were similar to those previously quantified using similar methods^25^. These discrepancies may be due, in part, to limitations in histology and immunohistochemistry which analyse small, arbitrary sections of the bulk tissue. In addition to collagen content, increased collagen type III may be connected with pelvic floor dysfunction and advanced utero-vaginal prolapse^56,57^ as it corresponds to increased extensibility in various soft biological tissues^19^. While no significant differences were identified in this study, large-diameter collagen fibres (potentially indicating collagen type I)^58^ and small-diameter fibres (potentially comparable to collagen III)^58^ appear to decrease and increase, respectively, with POP progression consistent with previous studies in other pelvic tissues^19,56,57,59–61^. Future work leveraging immunohistochemistry or protein quantification of type I and III collagen instead of PicroSirius Red would be highly valuable to further delineate relationships between USL microstructure and mechanical function.

This study demonstrated that USL mechanical behaviour was anisotropic, similar to prior studies^26^, but no significant differences in collagen content were identified between the two loading directions. However, a significant change in collagen orientation was observed between the POP III/IV and POP I/II groups suggesting remodeling of collagen fibres towards the perpendicular direction. This observation was supported by the smaller, but non-significant value of the anisotropy ratio in the POP III/IV group which may suggest decreased mechanical properties in the main *in vivo* direction and sustained properties in the perpendicular. It is possible that collagen fibres in the perpendicular direction are maintained throughout POP progression while collagen fibres in the main *in vivo* direction are potentially damaged due to loading or undergoing remodeling by resident cells. This is potentially supported by the lack of statistically significant differences in mechanical and histological properties in the perpendicular direction. These results may have important implications for designing synthetic or biomaterial grafts to bolster pelvic support, as prior work demonstrates that minimizing differences in graft and adjacent native tissue microstructure and mechanical function is critical to reduce graft complications^62^.

While no significant differences in peak stretch or tangent moduli were observed between the non-POP and POP I/II groups, the nonlinear model parameters corresponding to direction-dependent properties did increase compared to the non-POP. This may suggest extracellular matrix remodeling occurs with POP onset, but that those changes primarily correspond to decreases in extensibility within the low-strain regime that were not captured by the linearized tangent moduli values that describe the resistance to load in the high-strain regime. Interestingly, the average POP I/II stress-strain curves were shifted to the left, indicating the lowest extensibility compared to both the non-POP and POP III/IV groups. This may suggest that the USL remodels throughout POP progression and that different graft designs may be optimal for interventions at different POP stages. Further, the lack of significant correlation between collagen content and tangential modulus in the perpendicular direction suggests that other extracellular matrix components such as glycosaminoglycans or proteoglycans may also contribute to mechanical function. Contrary to our hypothesis, elastin content was not significantly altered nor significantly correlated to USL function, consistent with previous studies in the USL^57^ and contrasting those in the vaginal wall^63^. This may indicate that USL remodelling is driven by changes in loading following levator ani injury and/or collagen remodelling instead of elastic fibres. This may suggest that the structural processes during POP differ for each pelvic soft tissue, even though they are composed of similar proteins.

Additionally, there is a considerable amount of smooth muscle cells in the USL^19^, however, smooth muscle cells minimally contribute to passive biomechanical properties^64^ measured in this study. Future work is needed to characterize the contractile response of the USL with and without POP to better understand the contribution of smooth muscle cells. To accomplish this, samples must be immediately subjected to mechanical testing following surgery in media containing calcium, as opposed to flash frozen and tested in calcium-free media as performed in this study. Recent studies quantified the active properties of other pelvic tissues, such as the vagina, utilizing media with and without calcium, electric field stimulation, as well as various chemicals to induce either smooth muscle contraction or relaxation^62,65,66^. Smooth muscle cell contractility may play an important role in USL function, therefore future work is needed to understand how active mechanical properties may change in addition to the passive properties quantified in this study.

In addition to quantifying relationships between USL composition and mechanical function, potential correlations between established POP risk factors and USL composition were also examined. No significant correlations were identified, potentially due to the small sample size available herein. However, it is also worth noting that the non-POP control group did not show significant differences in BMI and parity compared to those with POP. This was surprising given the strong evidence that high BMI and multiparty are POP risk factors. This may be explained, in part, by the fact that 64% of adults in New Orleans were overweight or obese, with a higher obesity rate (31.4%) than the national rate (27.5%) in 2010^67^ and the obesity rate between the ages of 45–64 years was 42.9% in 2018 in Louisiana^68^. Hence, while our study was blind to BMI at the time of patient recruitment, the overall obesity rate in the study’s geographical location may have biased the control group.

This study was not without limitations. One limitation was the lack of racial diversity in the POP patient population. POP incidence in the African-American/Black population, however, is much lower than other racioethnic groups (Table 1). For example, Latina and white women have 4–5 times higher risk of symptomatic prolapse compared to African-American women^69^. Another limitation is that only a single portion of the USL was examined, yet the USL is heterogeneous^16,70–73^. To consistently and repeatably test a similar section of the USL across women, only the insertion sites of the USL were used in this study. Although this site is also the most easily accessible, the USL is a heterogeneous tissue and the biomechanical properties and composition/structure of the insertion site might differ from the main ligament^16,70–73^. The insertion site of USL by itself also exhibits greater anatomic variation, which may affect the extensibility and stiffness of the USL^71^. Further, the clinical significance of a small distal portion of the USL is uncertain, as it may not represent how the entire USL remodels and the mid-portion of the USL is used for POP repairs. Regardless, the information obtained herein provides a first step towards understanding how the USL remodels with POP progression in multiple directions and provides potential insights for direction- and stage-dependent treatments for POP. Another limitation was the use of histology to quantify USL composition instead of immunohistochemistry or assays to measure protein content. Further, while histology provided information on collagen orientation in the same region as the mechanical analysis, immunohistochemistry or protein quantification would have provided more specific information on multiple USL components and is needed in future studies. Lastly, while designed to detect statistically significant differences in mechanical properties, the study was underpowered due to the difficulty in obtaining samples from human patients and variability in the small sections of tissue – as a power analysis demonstrates that 54 samples would be required to detect statistical differences in USL composition.

Despite these limitations, this comparative study highlighted the importance of the biomechanical function of the USL in post-menopausal women at different stages of POP. In summary, the biomechanical properties in the main *in vivo* loading direction demonstrated that POP III/IV was more extensible than both non-POP controls and POP I/II. Further, the anisotropy of the USL changes with POP stage, which may be important for POP intervention design. Prior work demonstrated that incorporating biaxial characterization of pelvic floor muscle mechanical function improved the ability of finite element models to test structural and mechanical hypotheses of why and how failures of pelvic floor integrity occur^24^. In this study, we provide the first biaxial mechanical properties of the human USL with and without POP. Such information can be directly incorporated into existing finite element models to better understand how changes in USL function with POP stage may influence POP progression and surgical interventions.

## Data Availability

The datasets generated and the codes will be available to the public. Datasets will be stored in Dryad and the codes in GitHub, after manuscript has been accepted.
